# Antibody subclass skewing predicts enhanced ADCC activity in both natural infection and vaccination

**DOI:** 10.1186/1742-4690-9-S2-P82

**Published:** 2012-09-13

**Authors:** A Dugast, A Chung, Y Chang, A Licht, M Ackerman, G Alter

**Affiliations:** 1 Ragon Institute, Charlestown, MA, France; 2 Thayer School of Engineering, Dartmouth College, Hanover, NH, USA

## Background

The innate immune recruiting property of antibodies are elicited following an Fc/Fc-receptor interaction. We previously demonstrated that HIV-specific antibodies from elite controllers (ECs) robustly recruit NK cells to mediate antibody dependent cellular viral inhibition (ADCVI) compared to antibodies from chronic progressors. To gain further insights into the biophysical properties of EC antibodies that enable them to recruit Fc-effector functions so robustly, we performed at 24 dimensional analysis (including specificity, isotype, Fc-receptor affinity, and function) of the antibody profiles mounted in ECs and HIV-progressors to define the humoral signature(s) associated with most robust innate immune recruiting activity.

## Methods

A total of thirty donors were included in this study (10 untreated chronics, 10 treated chronics, 10 ECs). Anti-gp120, p24, gp41 and gp140 antibody binding titers were quantified by ELISA and a customized-multiplex HIV binding assay against HIV recombinant gp120, p24, gp41 and gp140. Fc-receptor affinity was analyzed by Biacore. ADCVI, ADCC, and ADCP were quantified as previously described.

## Results

While no differences were observed in the antibody binding titer between ECs and chronic progressors against gp120 or gp41, we showed that ECs exhibited a higher level of p24-specific antibodies, associated with robust ADCVI activity. Moreover, humoral responses in ECs were skewed toward IgG1 and IgG3 responses, compared to chronic progressors, that strongly predicted enhanced ADCVI activity. Similar skewing of antibody responses were observed in RV144 vaccinees, strongly suggesting that specific cues elicited within this vaccine trial may have resulting in the induction of highly potent innate immune recruiting antibodies similar to those found in ECs.

## Conclusion

Overall, ECs elicit a skewed humoral immune response marked by the preferential selection of HIV specific IgG1 and IgG3 antibody subclasses, that parallels the humoral immune responses observed in RV144 vaccinees. Therefore, characterizing these unique antiviral capacities may provide critical information on humoral responses that could potentiate vaccine-induced responses.

